# Research on UAV dynamic frame rate adaptation and multi-feature fusion network optimization in intelligent monitoring of animal husbandry

**DOI:** 10.1371/journal.pone.0331850

**Published:** 2025-09-22

**Authors:** Wei Luo, Lin Li, Xinping Luo, Quanqin Shao, Ruiyin Tang, Ke Liu, Xuqing Li, Xiaohuang Liu, Qi Wang, Dongyue Ren, Dongliang Wang

**Affiliations:** 1 North China Institute of Aerospace Engineering, Langfang, China; 2 Aerospace Remote Sensing Information Processing and Application Collaborative Innovation Center of Hebei Province, Langfang, China; 3 National Joint Engineering Research Center of Space Remote Sensing Information Application Technology, Langfang, China; 4 Comprehensive Survey Command Center for Natural Resources, China Geological Survey, Beijing, China; 5 Key Laboratory of Coupling Process and Effect of Natural Resources Elements, Ministry of Natural Resources, China; 6 Key Laboratory of Land Surface Pattern and Simulation, Institute of Geographic Sciences and Natural Resources Research, Chinese Academy of Sciences, Beijing, China; 7 University of Chinese Academy of Sciences, Beijing, China; 8 Hebei Geological Surveying and Mapping Institute, Langfang, China; University of Agriculture Faisalabad, PAKISTAN

## Abstract

Precision livestock farming, particularly the collective rearing of animals, remains a pivotal area of focus within agricultural research. However, tracking group-raised animals under conditions of poor lighting, occlusion, and complex outdoor environments continues to pose significant challenges. Due to the intricacies of these conditions, existing methodologies frequently encounter reduced tracking accuracy, decelerated processing rates, and recurrent failures amid occlusion and drift. In response to these challenges, this study introduces SiamCMR, a sophisticated RGB-Thermal (RGBT) object tracking framework tailored for the prolonged observation of group-raised Holstein cows. Constructed upon a dual-stream Siamese network architecture, SiamCMR incorporates innovative feature fusion techniques to deliver robust, real-time tracking capabilities. The framework utilizes a Complementary Coupled Feature Fusion (CCFF) module that merges semi-shared convolutional filters with adaptive sigmoid weighting to efficaciously amalgamate modality-specific features derived from RGB and thermal inputs. To further refine the fusion quality under diverse illumination conditions, we have developed a Multimodal Weight Penalty Module (MWPM), which selectively emphasizes informative channels via batch normalization scaling and feature variance analysis. The framework’s resilience to occlusions and drift is enhanced through the integration of reinforcement learning. In experimental evaluations using our proprietary dataset, SiamCMR maintained real-time processing at 135 frames per second (FPS), achieving 81.3% precision (PR) and 58.2% success rate (SR). When compared to the baseline Siamese tracker, SiamFT, which recorded 76.5% PR, 56.2% SR, and 45 FPS, our approach exhibited improvements of 4.8% in PR, 2.0% in SR, and a threefold increase in processing speed, thereby enhancing both tracking accuracy and robustness. Moreover, the system’s efficacy has been corroborated through successful implementations on a UAV platform in real-world ranch settings. Results from ablation studies under severe occlusions, light interference, low illumination, and low temperatures validate the effectiveness of the primary components. This research delineates an innovative real-time cattle-tracking solution that augments pasture management by facilitating precise monitoring of cow positions, behaviors, and health, ultimately optimizing feeding strategies and enhancing milk quality and safety.

## 1. Introduction

Agriculture serves as the bedrock of human society, underpinning global food security, livestock production, and the economic vitality of rural areas. Concurrently, it is experiencing a profound transformation driven by the global wave of digitalization. The adoption of intelligent monitoring technologies in animal husbandry is now critical, enhancing productivity, ecological sustainability, and animal welfare. Dairy farming, a fundamental component of traditional agricultural practices, plays an instrumental role in the economic prosperity and development of rural communities [1]. This significance is underscored by research, such as that conducted by Olatinwo et al. [2], which illustrates the beneficial effects of active agricultural engagement on rural living standards. In agriculture, various kind of modern and traditional agricultural activities are being practiced to rural economies and nations food security [3,4]. The integration of intelligent monitoring systems into livestock farming is particularly vital for improving productivity, sustainability, and welfare [5,6]. Within this domain, Holstein cows, noted for their superior milk production [7], contribute significantly to rural economies through breeding programs aimed at enhancing genetic quality and productivity [1]. Tracking Holstein cows enables monitoring of movement, health, and social behaviors, which aids in early detection of diseases and environmental stressors, ultimately improving fertility and milk yield. Presently, the methods employed for livestock tracking include manual monitoring [8], tattooing [9], ear tagging [9], branding [10–12], and collaring [13]. However, these techniques often demand extensive manual labor, can disrupt animal behavior, and escalate operational costs, rendering them impractical for large-scale operations. As the scope of Holstein cow breeding expands, the industry faces increasing labor demands and exacerbating labor shortages. In this context, smart animal husbandry emerges as a transformative advancement over traditional livestock farming methods. By integrating modern information technology and automation, it facilitates highly efficient, intelligent, and precise livestock management, as evidenced by recent advancements in precision dairy management [14] and health monitoring systems [15]. These technologies allow for the real-time and accurate tracking of Holstein cows and, through the detailed perception and analysis of individual data, promote the management of smart farms and the establishment of animal-friendly environments. Among the various technological innovations in smart animal husbandry, drones play a pivotal role. They significantly reduce human intervention in contrast to traditional monitoring methods and offer substantial benefits in terms of reducing risks and operational costs [16,17].

The field of visual target tracking predominantly relies on deep learning (DL) and correlation filtering-based (CF) techniques [18]. In recent years, the integration of UAV technology with DL has become increasingly prevalent in wildlife monitoring. This synergy allows for the analysis of extensive datasets in real-time, addressing challenges such as low detection accuracy, high model complexity, and suboptimal performance encountered with conventional machine learning (ML) methods [19–21]. This combination has proven particularly effective in overcoming issues related to time consumption and inefficiency in livestock management on expansive farms. Nevertheless, UAVs frequently operate in adverse conditions, such as darkness and fog, which compromise visible light imaging and exacerbate noise levels, thus impeding the application of traditional algorithms. In these scenarios, thermal imaging emerges as a superior alternative by detecting infrared signals emitted from animal body heat, thereby facilitating consistent target identification and trajectory tracking in conditions of low visibility. By the complementary strengths of infrared and visible light, a UAV equipped with the appropriate technology can perform rapid and precise monitoring under challenging conditions. To date, various RGB-T tracking methods, including SiamFT, MANet++, and mfDiMP, have been extensively utilized. Despite their widespread deployment, these methods often falter in environments characterized by significant appearance changes, cluttered backgrounds, rapid target movements, and frequent occlusions, underscoring the necessity for ongoing research and enhancement. Accordingly, this study conducts a comprehensive and detailed analysis of each component involved. The principal contributions of this study are as follows:

1. We introduce a novel RGB-T tracking framework, SiamCMR, predicated on a dual-stream Siamese neural network architecture, which is specially tailored for real-time monitoring of Holstein cows in pasture settings. The framework incorporates multiple collaborative modules to ensure robust and efficient tracking under a variety of demanding conditions. Experimental evidence confirms the framework’s ability to sustain high tracking precision while operating in real-time (135 FPS), even in scenarios plagued by severe occlusions, fluctuating lighting conditions, and thermal disturbances, thereby proving its suitability for large-scale, UAV-based intelligent ranch surveillance.
2. The framework integrates a Complementary Coupling Feature Fusion (CCFF) module designed to extract akin features, diminish inter-modal discrepancies, and augment feature fusion. Additionally, we introduce a multi-modal adaptive weight penalty (MAWP) module that integrates a weight contribution factor to assess the significance of each modal feature. This module utilizes batch normalization scale factors and standard deviations to highlight the relevance of weights, thereby offering more precise and focused feature information to improve tracking performance.
3. Building upon the SiamCMR framework, we have developed a dynamic template update strategy predicated on tracking outcomes, both successful and unsuccessful. This sophisticated mechanism allows the model to adjust to variations in a cow’s appearance—such as pose shifts, angular modifications, and occlusions—by autonomously updating the reference template. This implementation significantly curtails tracking drift that typically results from template aging, thereby enhancing long-term tracking stability.
4. We introduce a novel reinforcement learning-based dynamic decision mechanism that exploits similarity score maps produced by Siamese networks to refine tracking strategies. By delineating specific states, actions, and reward functions, and by integrating aspects such as target motion direction and historical trajectory data, our methodology facilitates intelligent livestock tracking. Tailored expressly for livestock monitoring contexts, this approach epitomizes innovation in RGB-T multimodal tracking frameworks through its application-specific optimization at the score map level.

The organization of this study is as follows: Section 2 offers an exhaustive review of pertinent methodological developments in the field. Section 3 details the data acquisition process and elaborates on the implementation specifics. Sections 4 and 5 explore the presentation and analysis of the experimental results, respectively. Section 6 provides the conclusion of the study.

## 2. Related work

### 2.1 Current status of MOT

Multiple Object Tracking (MOT) has emerged as a vital and complex task within the domain of computer vision [22]. In the realm of intelligent animal husbandry, the deployment of vision-based MOT algorithms has notably expanded, solidifying a promising research trajectory. Among the solutions addressing the challenges associated with tracking multiple objects, methodologies such as Kalman filters [23,24], particle filters [25,26], Multi-Hypothesis Tracking (MHT) [27,28], Joint Probabilistic Data Association Filter (JPDAF) [29–31], Gaussian-Based MOT (GBMOT) [32,33], and Random Finite Set (RFS) filters [34] have been widely adopted. These multi-object trackers are designed to monitor an indefinite number of entities within a predefined category set [36], primarily functioning on the principle of connecting detection points across successive frames [37,38]. Bea et al. [39] devised a Siamese neural architecture to derive discriminative metrics between object pairs. FairMOT [40] integrates the tasks of object detection and appearance embedding using a unified backbone architecture, thereby improving the accuracy of object association. As the applications of MOT become increasingly multifaceted, the systems face numerous challenges including frequent occlusions, the initiation and cessation of object trajectories, and the occurrence of objects within the same category exhibiting similar appearances [35]. To overcome these challenges, we propose a reinforcement learning model tailored to a similarity score map generated by the Siamese network, specifically defining states, actions, and rewards to effectively address occlusion issues.

### 2.2 Status of RGBT tracking

Recent advancements in RGB-T fusion tracking algorithms have been classified into five primary categories: traditional approaches, SR-based methods, graph-based techniques, correlation-based filters, and DL-based strategies. Among these, deep learning-based strategies have garnered significant research interest. Numerous trackers that utilize deep features, such as SiamCDA, have been developed [41–43]. Typically, RGB-T trackers evolve from RGB trackers; for example, they have served as baseline models in multiple studies [41,42]. Notably, Zhu et al. [41] pioneered a network that integrates features from all layers and modes before pruning them to reduce noise and redundancy. Li et al. [42] introduced an innovative multi-adapter framework that separately trains on modality-shared, mode-specific, and instance-aware target representations. Additionally, Zhang et al. [43] utilized DiMP [44] as a core tracker and investigated various fusion processes at multiple levels to identify the most efficacious fusion architecture. To enhance the robustness of cross-modal tracking, several recent studies have devised novel fusion strategies that better align thermal and visible features. For instance, Superthermal [45] investigates thermal feature transformation to augment compatibility with visual domain representations, thus enabling more precise matching under challenging conditions such as low light or fog. Similarly, EMAT [46] offers an efficient fusion strategy based on optimized multi-head attention, dynamically adjusting modality contributions in response to variations in target appearance. These innovations are particularly advantageous for livestock tracking, where targets frequently encounter occlusion and deformation. Furthermore, multi-level fusion architectures, like those examined by Wang et al. [47], integrate semantic and texture-level cues to enhance image robustness and quality across modalities. This integration proves valuable when RGB or thermal channels are partially degraded. Despite these advancements, many existing methods do not fully exploit the critical advantages arising from the discrepancies between visible and thermal infrared modalities, which are essential for achieving comprehensive modality fusion. Moreover, the issue of tracker drift, caused by changes in target appearance, is often overlooked despite its critical impact on successful tracking. To mitigate these limitations, we propose a reinforcement learning-based dynamic template update strategy within the SiamCMA framework. By continuously refining the template based on feedback from tracking performance, our system adeptly adapts to appearance variations, effectively reducing the risk of tracking drift.

### 2.3 Current status of dairy cow monitoring efforts

The current landscape of dairy cow monitoring is marked by significant technological advancements and accompanying challenges. Recent developments have seen the emergence of non-contact tracking methods that utilize machine vision technology, presenting new opportunities for dairy cow surveillance. These methods effectively address the limitations associated with traditional contact-based tracking systems, as documented by researchers such as Koniar [48], Bergamini [49], and Gao [50]. Prior research, including studies by Sun [51] and Xiao [52], has developed tracking algorithms originally designed for pigs. These algorithms require meticulous design of low-level features, are labor-intensive, and necessitate stringent environmental conditions, rendering them impractical for direct application in dairy cow monitoring in real-world scenarios. CNNs have become the predominant technology for livestock monitoring. Research by Boogaard et al. [53] and Gao et al. [54] has significantly advanced the automated extraction of semantic features at both low and high levels, thereby streamlining the monitoring process for dairy cows. Zhang et al. [55], Zhang et al. [56], and Tu et al. [57] have successfully adapted modified YOLO networks and the DeepSort algorithm, originally applied to beef cattle and pigs, for MOT purposes, and these methodologies are equally applicable to dairy cows. However, complex environmental factors such as occlusion among cows and variable lighting conditions in practical settings pose significant challenges. These issues can lead to the generation of numerous low-scoring detection boxes, which may undermine tracking performance. Tassinari et al. [58] implemented a YOLOv3 model to identify and track individual dairy cows, but their study was limited to just four subjects. In contrast, Guzhva et al. [59] introduced a CNN-based method for continuous tracking and labeling of individual dairy cows, offering a novel approach to monitoring. Current research predominantly focuses on monitoring small groups of cows within controlled indoor environments. The complexities increase with the size of the herd, making manual identification and annotation increasingly burdensome. These challenges highlight the pressing need for the development of enhanced methodologies in the contemporary surveillance of dairy cows.

## 3. Materials and methods

### 3.1 Data acquisition

#### 3.1.1 Research area and subject selection.

**Fig 1:**
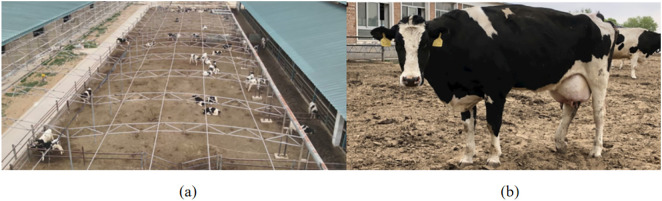
Overview of the study area and selected study subjects. **(a)** Aerial image of the study area captured by UAV. **(b)** Selected study subjects.

The research was conducted at Youzhi Ranch, located in the Luquan District of Shijiazhuang City, Hebei Province, China. This site was chosen as the primary location for data collection and flight verification. Video recordings captured Holstein cows that exhibited well-developed hindquarters, wedge-shaped body profiles, distinct black-and-white markings, and characteristic white spots on the lower legs and tail. The designated sampling zone was outlined at an interval of 10 meters within aerial photographs (Fig 1a). The primary aim of the study was to observe the natural behaviors of Holstein cows without causing disturbances or making environmental changes. Following multiple sampling events, subjects were chosen based on criteria such as large body size, symmetrical shape, thin skin, slender skeletal structure, and minimal subcutaneous fat (Fig 1b).

#### 3.1.2 Acquisition platform.

The experimental framework utilized the DJI Mavic 3T thermal imaging drone, a robust industrial UAV characterized by its compact design. The drone’s specifications include a bare weight of 920 grams and fuselage dimensions of 347.5 × 283 × 107.7 mm (length × width × height). It provides a maximum flight duration of 45 minutes, an operational range of 32 kilometers, a payload capacity of 1050 grams, and a service ceiling of 6000 meters, making it well-suited for a wide range of UAV applications. The platform includes three integrated camera systems: wide-angle, telephoto, and thermal imaging. The thermal camera offers a temperature detection range from −20°C to 150°C, with a measurement accuracy of ±2°C at the higher end of this range. Data processing was performed using the NVIDIA Orin NX computing platform, the specifications of which are detailed in Table 1.

**Table 1:** Nvidia Orin NX parameters table*.

CPU	CPU Frequency	Display Memory	Computational Performance
Arm Cortex-A78AE	2GHz	16GB	100TOPS

#### 3.1.3 Data preparation.

**Fig 2:**
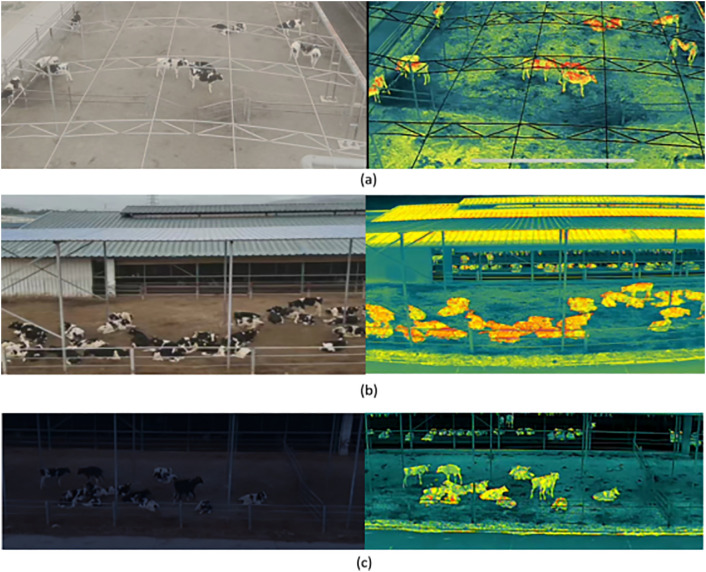
Representative environmental conditions during data collection. **(a)** Moderate fog, **(b)** Natural occlusions, **(c)** Low-light nighttime condition.

To enhance the practical utility of the dataset, data collection was conducted from April to May 2024 across several Holstein cow farms in the Luquan District, Hebei Province. The UAV was equipped with an array of cameras including wide-angle, telephoto, and downward-facing thermal imaging capabilities, enabling the synchronized capture of RGB and thermal videos without necessitating post-processing. Recordings were executed during three distinct daily periods—morning, midday, and evening—to capture a diverse range of lighting and temperature conditions. A variety of environmental settings were purposefully selected, encompassing clear daylight, moderate fog (Fig 2a), low-light nighttime conditions (<5 lux) (Fig 2c), and scenarios featuring natural occlusions such as fences or overlapping cows (Fig 2b). The drone maintained altitudes between 10–15 meters and a maximum speed of less than 1 m/s to ensure the stability of the imagery. After excluding footage that was distorted or lacked the target, a collection of 75 high-quality MP4 videos (1920 × 1080 @ 25 fps), each spanning 25–35 minutes, was compiled.

To establish robust ground truth, keyframes were manually extracted and annotated to depict typical behaviors such as feeding, walking, resting, and excretion, as well as challenging conditions like occlusion and deformation. Annotations were performed independently by two trained experts using the LabelImg and VIA tools, with discrepancies being resolved through a review conducted by a third annotator. The final labels were stored in both YOLO and Pascal VOC formats, with each object instance tagged with one or more of seven difficulty attributes as per the Generic Thermal Object Tracking (GTOT) standard: occlusion (OCC), large scale variation (LSV), fast motion (FM), low illumination (LI), thermal crossover (TC), small object (SO), and deformation (DEF). The RGB–thermal paired image dataset was subsequently divided into training (6,300), testing (1,800), and validation (900) subsets, adhering to a 7:2:1 ratio to ensure balanced evaluation and effective model generalization.

### 3.2 Overall technical framework

**Fig 3:**
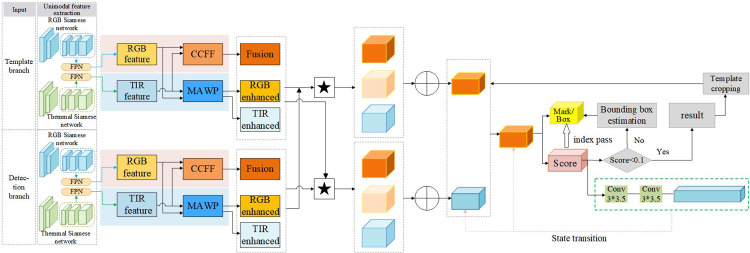
The architecture of the RGBT multi-tracking framework.

This section delineates the proposed RGBT tracking framework based on a Siamese neural network, with the overall architecture depicted in Fig 3. The framework integrates four core modules. Initially, the SiamCMA module undertakes unimodal feature extraction independently on both RGB and thermal infrared (TIR) images. Subsequently, the CCFF module enhances and amalgamates the two modalities through both coupled and uncoupled convolutional filters. Following this, MWPM allocates weights to features according to the significance of each channel, effectively minimizing irrelevant or redundant information. Lastly, a reinforcement learning module dynamically refines tracking decisions based on similarity score maps and historical motion patterns, simultaneously facilitating adaptive template updates. Through the integrated operation of these modules, the system ensures robust and efficient multi-object tracking capabilities in complex environments characterized by occlusion, low illumination, and thermal crossover.

### 3.3 Multi-modal adaptive tracking framework with reinforcement learning

#### 3.3.1 Dual-stream siamese network for unimodal feature extraction.

The tracking framework incorporates a dual SiamCMA architecture to independently process RGB and thermal inputs. Although both networks maintain structural similarity, they utilize distinct parameters tailored to enhance feature extraction for each respective modality. Each Siamese neural subnetwork comprises two branches: the template branch and the detection branch. These branches are structurally identical and share parameters. The primary function of the template branch is to extract features from the template patch.

**Fig 4:**
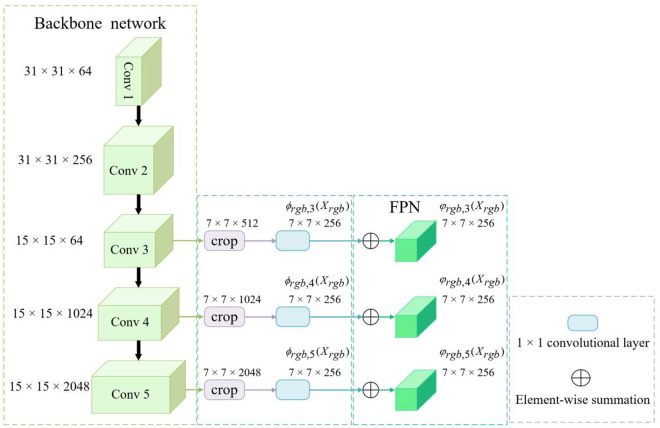
Schematic depiction of the template branch of the RGB SiamCMA, consisting of a backbone network merged with a FPN.

In the development of the feature extraction module for unimodal analysis, the architectural framework of the RGB branch of the SiamCMA was employed as a reference. This decision was prompted by the consistent architecture observed across all four branches of the dual-stream SiamCMA network. As depicted in Fig 4, the RGB SiamCMA template branch integrates a ResNet50 backbone with a Feature Pyramid Network (FPN).

To address the performance constraints inherent in Siamese trackers, our investigation employs a methodology advocated by Li et al. [65], which incorporates the advanced deep learning architecture ResNet50 [66] as the primary backbone network. This choice is based on the premise that deeper networks can significantly enhance tracking performance. As depicted in Fig 4, the architecture we propose features several critical modifications to the standard ResNet50 design. A notable innovation in our approach involves eliminating the downsampling operations in the final two convolutional blocks (conv4 and conv5) by setting the stride to 1. This adjustment improves the resolution of the feature maps, thereby facilitating a more precise capture of fine-grained details within the template patch. Additionally, we incorporate dilated convolutions in these blocks with dilation rates of 2 and 4 for conv4 and conv5, respectively. This strategy increases the receptive field while maintaining feature map resolution. To further enhance efficiency, a 1 × 1 convolutional layer is added after each of the last three blocks (conv3, conv4, and conv5), reducing the channel count to 256. For computational optimization, feature extraction is confined to the central 7 × 7 region of the template, as empirical evidence suggests that this area harbors critical target features while minimizing computational demands. We intentionally omit features from the first two blocks (conv1 and conv2) due to their tendency to introduce noise, which impairs tracking performance.

The template (or detection) patch processes information through a three-tiered feature extraction mechanism within the backbone network. At the primary level, the extracted features concentrate on visual components such as edges, corners, colors, and shapes, which are crucial for precise object localization. Conversely, features at a higher level capture more semantic details essential for object identification. Integrating features from both levels markedly improves the PR of object tracking. To augment the feature extraction capabilities of the backbone network, an FPN was integrated into its last three blocks. This modification enables the integration of cross-level features derived from the hierarchical layers labeled $\varphi_{\text{rgb},\text{i}}\left({\text{x}}_{\text{rgb}}\right)(\text{i}=\text{3},\text{ 4},\text{ 5})$}. In our network configuration, the $\varphi_{\text{rgb},\text{i}}\left({\text{x}}_{\text{rgb}}\right)(\text{i}=\text{3},\text{ 4},\text{ 5})$} features, which maintain consistent spatial resolutions and channel counts, were amalgamated using a top-down approach to generate the FPN output for each corresponding layer, represented as $\varphi_{\text{rgb},\text{i}}\left({\text{x}}_{\text{rgb}}\right)(\text{i}=\text{3},\text{ 4},\text{ 5})$}. The resulting FPN output was then designated as the output features of the template branch in the RGB SiamCMA, identified as $\phi_{\text{rgb}}\left({\text{x}}_{\text{rgb}}\right)$ = {$\varphi_{\text{rgb},\text{i}}\left({\text{x}}_{\text{rgb}}\right)(\text{i}=\text{3},\text{ 4},\text{ 5})$}. Similarly, the output features of the detection branch in the RGB SiamCMA, denoted as $\phi_{\text{rgb}}\left({\text{z}}_{\text{rgb}}\right)$ = {$\varphi_{\text{rgb},\text{i}}\left({\text{z}}_{\text{rgb}}\right)(\text{i}=\text{3},\text{4},\text{5})$}, were derived using a similar summation strategy. Upon securing these features from the respective branches, the CCFF module was devised to effectively fuse the features extracted from the dual-mode SiamCMA.


$$\;\phi_{\text{rgb},\text{5}}\left(x_{\text{rgb}}\right)=\varphi_{\text{rgb},\text{5}}\left(x_{\text{rgb}}\right)$$ (1)

$$\phi_{\text{rgb},\text{4}}\left(x_{\text{rgb}}\right)=\varphi_{\text{rgb},\text{4}}\left(x_{\text{rgb}}\right)⊕\phi_{\text{rgb},\text{5}}\left(x_{\text{rgb}}\right)$$ (2)

$$\phi_{\text{rgb},\text{3}}\left(x_{\text{rgb}}\right)=\varphi_{\text{rgb},\text{3}}\left(x_{\text{rgb}}\right)⊕\phi_{\text{rgb},\text{4}}\left(x_{\text{rgb}}\right)$$ (3)

#### 3.3.2 Complementary coupling feature fusion module (CCFF).

**Fig 5:**
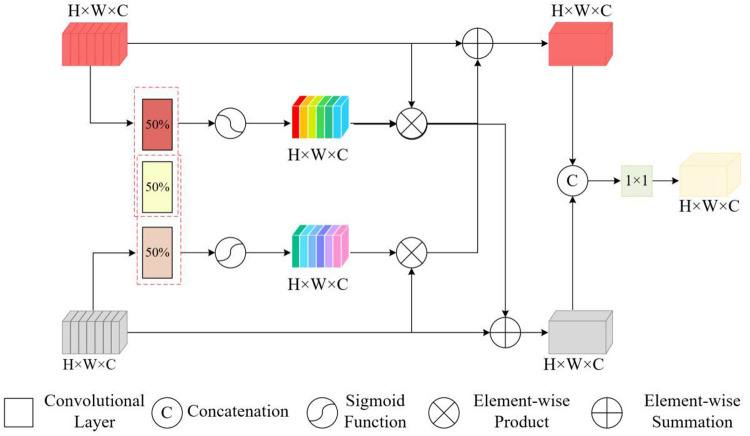
CCFF module, adaptively integrating RGB and thermal infrared features via coupled and uncoupled convolutional filters.


Building on the approach proposed in the study by [40], which introduced coupled convolutional filters to enhance modality interaction in RGB-T tracking, our work expands this concept through the design of a CCFF module. This module not only facilitates enhanced integration of cross-modal features but also incorporates adaptive weighting to augment tracking robustness under challenging conditions. Our CCFF module employs coupled filters within the convolutional layers at a coupling ratio of 0.5. This configuration enables the filters to concurrently update the weights of both visible and thermal infrared features, thus optimizing cross-modal feature integration. As depicted in Fig 5, the CCFF module integrates coupled convolutional filters to concurrently enhance RGB and TIR features. Each convolutional layer is equipped with both coupled and uncoupled filters, each having a kernel size of 3 × 3. The coupled filters undergo updates twice per iteration, generating weight maps that regulate the extent of information exchange between modalities. These weights are normalized to a range of [0,1] using a sigmoid function, which facilitates the enhancement across modalities. Subsequently, the enhanced features are concatenated and processed through a 1 × 1 convolutional layer to standardize the channel dimensions, thereby creating a robust fused representation. For instance, within the template branch, the weight maps were derived as follows:

$$W_{r}=\sigma \left(\text{conv}\left(\phi_{\text{rgb}}\left(x_{\text{rgb}}\right),\theta_{1}\right)\right)$$ (4)

$$W_{t}=\sigma \left(\text{conv}\left(\phi_{t}\left(z_{t}\right),\theta_{2}\right)\right)$$ (5)

where conv(*,θ) signifies a convolutional layer characterized by parameter θ, encompassing both uncoupled and coupled filters with identical parameters. Here, σ(·) denotes the sigmoid function. Following the computation of the weight map, both visible light and thermal infrared features are enhanced through cross-modal interactions involving ${\text{W}}_{\text{r}}$ and ${\text{W}}_{\text{t}}$.


$$\phi_{r}^{\prime }\left(x_{\text{rgb}}\right)=\phi_{\text{rgb}}\left(x_{\text{rgb}}\right)+\phi_{t}\left(z_{t}\right)⊗W_{t}$$ (6)

$$\phi_{t}^{\prime }\left(z_{t}\right)=\phi_{t}\left(z_{t}\right)+\phi_{\text{rgb}}\left(x_{\text{rgb}}\right)⊗W_{r}$$ (7)

Subsequently, the enhanced features $\phi_{\text{r}}^{\prime }\left({\text{x}}_{\text{rgb}}\right)$ and $\phi_{\text{t}}^{\prime }\left({\text{z}}_{\text{t}}\right)$ were obtained, and their fusion was accomplished by concatenation. A 1 × 1 convolutional layer was then utilized to integrate the channel information, resulting in a combined feature$\;{\text{z}}_{\text{f}}$.


$$z_{f}=\text{conv}\left(\text{cat}\left(\phi_{r}^{\prime }\left(x_{\text{rgb}}\right),\phi_{t}^{\prime }\left(z_{t}\right)\right),\theta_{3}\right)$$ (8)

#### 3.3.3 Multi-modal weight penalty module (MWPM).

The MWPM algorithm assesses the characteristics of both modalities in a unified manner and assigns weights to the deep features based on this comprehensive evaluation. To compute the attention weights, MWPM utilizes scaling factors obtained from BN [28], which function to suppress less significant features by incorporating regularization terms. These scaling factors, within the channel attention submodule, quantify the degree of variation in each channel, thus indicating the channels’ relative significance.


$$\text{BN}\left(x_{i}\right)=\gamma {\hat{\text{x}}}_{i}+\beta$$ (9)

Here, ${\hat{\text{x}}}_{\text{i}}$ denotes the normalized eigenvalue, while γ and β serve as adjustable reconstruction parameters. These parameters facilitate the network’s ability to adaptively restore the feature distribution akin to that of the original network. The application of the scaling factor, derived from the variance observed in BN, follows a specific rationale: a higher variance indicates a more substantial change in the channel, suggesting that the information within that channel is of greater importance, whereas channels with less variance carry less critical information.


$$C_{\text{out}}=\frac{\gamma_{c_{i}}}{\sum_{j=0}^{c}\gamma_{\text{ci}}}\left(\text{BN}\left(C_{\text{in}}\right)\right)$$ (10)

$$\;{\text{S}}_{\text{out}}=\frac{\gamma_{s_{i}}}{\sum_{j=0}^{N}\gamma_{\text{si}}}\left(\text{BN}\left(S_{\text{in}}\right)\right)$$ (11)

In Equation (10), the input feature, denoted ${\text{C}}_{\text{in}}$, undergoes processing to produce the output feature, labeled ${\text{C}}_{\text{out}}$, with $\gamma_{{\text{c}}_{\text{i}}}$ acting as the scaling factor for each respective channel. By employing a consistent normalization strategy across all pixels within the spatial domain, we can establish the spatial attention mechanism as delineated in Equation (11).

**Fig 6:**
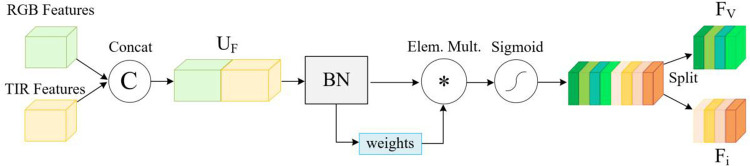
The MWPM connects the deep features of the RGB mode and the thermal infrared mode, and then assigns weights to all channels.


As depicted in Fig 6, the RGB and TIR features are combined along the channel dimension to create a cohesive representation of both the template and search features within the channel attention module.

$$U_{F}=cat(F_{RGB},F_{T})$$ (12)

Subsequently, ${\text{U}}_{\text{F}}$ is penalized with characteristic weights, and the resulting output is expressed as follows:


$$\left(F_{v},F_{i}\right)=\text{Split}\left(\delta \left(\frac{\gamma_{c_{i}}}{\sum_{j=0}^{c}\gamma_{\text{ci}}}\left(\text{BN}\left(U_{F}\right)\right)\right)\right)$$ (13)

The function δ(·) denotes the sigmoid activation, while “Split” refers to the division of features according to channel size. In the integration of the two submodules, channel attention is initially applied, followed by spatial attention. The channel attention mechanism reduces the prominence of less pertinent feature channels, while the spatial attention mechanism aims to attenuate background noise.

Following the processing by the attention module, the enhanced feature images of the template and search images are further processed by MWPM. Initially, the algorithm correlates the two branches of visible light to produce the response map R2. A parallel correlation is conducted on the two branches of thermal infrared, resulting in another response map, denoted R3. Through a tri-layer channel compression process, certain features are extracted, followed by the fusion of R1*, R2*, and R3*. Additional channel compression subsequently leads to the final feature map, represented as R-final (25 × 25 × 256).


$$R-\text{final}=\text{Cat}\left(R1^{⋆},R2^{⋆},R3^{⋆}\right)$$ (14)

#### 3.3.4 Reward-driven learning mechanism.

In this section, we discuss the application of reinforcement learning for optimal feature selection. By leveraging the feature map generated by the score branch of a Siamese-based tracking model, the complexity of the input image was effectively reduced. This reduction was achieved through the maximization of input simplification directed towards the reinforcement learning model. Subsequently, the features extracted from the score map were utilized as input variables for the reinforcement learning model.

(1) Action

The state was defined by utilizing both the score map and the movement direction of the object. The score map was modified by integrating the object’s movement direction as a weighting factor. Upon receiving the target image, denoted as z, and the search image, referred to as x, as inputs, the score branch identified the position with the highest score. Action A is delineated as a 9-dimensional vector, comprising the central position as defined by Equation 15 and the eight adjacent positions within the same channel as delineated by Equation 16. Here, a₀ = (m − 1, n − 1), a₁ = (m − 1, n),..., a₄ = (m, n),..., a₇ = (m + 1, n), a₈ = (m + 1, n + 1).


$$m,n=\text{argmax}\left(F_{\text{score}}(z,x)\right)$$ (15)

$$A=[\begin{array}{c} a_{0}\\ a_{1}\\ \vdots \\ a_{7}\\ a_{8} \end{array}]$$ (16)

The output feature map produced by the score branch was processed using the softmax function to derive score probabilities, which were then employed to select an action for training. The chosen index was subsequently inputted into either a bounding box or a mask branch, where a bounding box was predicted to delineate the object’s location corresponding to that index.

(2) Status

As defined by Equation 17, the term “State S” encapsulates a combination of a score map and a vector indicating the object’s movement direction.


$$S=\left(F_{\text{score}},\text{b}{\text{b}}_{d}\right)$$ (17)

The score map serves to condense the information present in the original image. The orientation of the object was inferred by estimating the bounding box based on the previously selected action. The motion direction, represented by a unit vector, was derived from the position of the bounding box. The trajectory of movement over the previous 10 frames up to the current frame was formulated as a vector. In Equation 17, ${\text{F}}_{\text{score}}$ denotes the similarity score map, and $\text{b}{\text{b}}_{\text{d}}$ refers to the vector encapsulating the movement direction of the bounding box. Consequently, the state included the location details of the area that exhibited the highest similarity to the target.

(3) Reward

In numerous tracking algorithms that incorporate offline learning, a tracking error at the onset of a sequence may precipitate cumulative errors, ultimately causing the tracker to erroneously follow an incorrect object or background element. The reward function within our model was established based on the Intersection over Union (IoU) between the actual bounding box ($\text{b}{\text{b}}_{\text{G}}$) and the predicted bounding box ($\text{b}{\text{b}}_{\text{E}}$) observed in the final frame of the sequence. This design aims to foster effective learning even when the object is partially occluded (PO). Consequently, the reward was structured as described in Equation (18), paralleling the ADNet approach, where an IoU of 0.7 or greater is achieved at the conclusion of the sequence.


$$R_{L}=\{\begin{array}{cc} 1, & \text{ifIoU}=\frac{\text{b}{\text{b}}_{G}\cap \text{b}{\text{b}}_{E}}{\text{b}{\text{b}}_{G}\cup \text{b}{\text{b}}_{E}}>0.7\\ -1, & \text{otherwise} \end{array}\}$$ (18)

(4) State Transition

The process of selecting an action within a particular state initiates a transition to the next state. This state transition is dictated by a function that is contingent on the action selected, as demonstrated in Equation (19).


$$s_{t+1}=f_{\text{transition}}\left(s_{t},a_{t}\right)$$ (19)

(5) Realization

**Fig 7:**
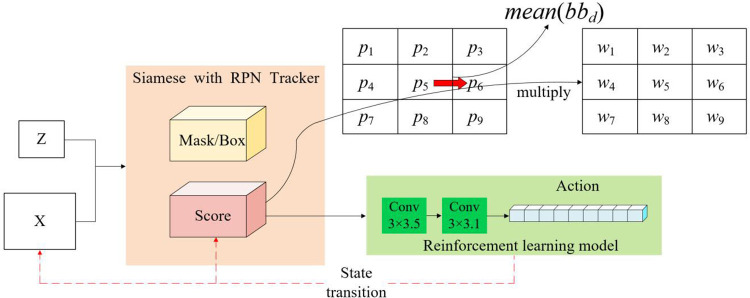
Implementation of the reinforcement learning-based dynamic tracking decision mechanism integrating score maps and motion direction.


Within the architectural framework of our reinforcement learning model, feature extraction is executed through two layers of 3x3 convolution applied to the score map. This is followed by a dense layer that produces outputs corresponding to nine predefined actions. Over a period of 10 frames, the average movement direction, denoted as $\text{b}{\text{b}}_{\text{d}}$, is calculated as the mean vector of directional movements. The decision-making mechanism involves a point-wise multiplication of the weight vectors ${\text{w}}_{\text{k}}$ (k ∈ [0,8]) with the average direction across the score map to select an action, as illustrated in Equation (20) of Fig 7.

$$a=M_{\text{RL}}\left(F_{\text{score}}\times w_{k}\right)$$ (20)

In Fig 7, when the prevailing movement direction is to the right, the algorithm assigns an enhanced weighting to this rightward direction (${\text{W}}_{\text{k}}$). This decision leverages an average of directional data over the preceding 10 frames to compute actions on the score map. For assessing tracking failures, the method of dynamic template switching is employed, which considers the tracking output that achieved the highest score in the previous frame and uses it as the template for the current frame. This template is then correlated with the search image in the correlation layer of the target image. The tracking result that receives the highest score during this process is designated as the definitive output. This dynamic switching method is designed to activate after several frames, thereby accommodating the initially low probability of target loss and minimal target variation.

(6) Train

In instances of tracking failure, the dynamic template switching strategy involves selecting the highest scoring tracking result from the preceding frame as the new reference template. This template is then correlated with the search image in the correlation layer to pinpoint the target. The highest scoring result is subsequently adopted as the definitive output. Given that the initial stages of tracking typically present minimal risk of target loss and that significant changes to the target are unlikely, this method is implemented at specific intervals following the commencement of tracking. The experimental conditions were established using a randomly selected sequence, and the learning parameters were adjusted based on the rewards obtained from the final frame of that sequence.

In the final frame of the sequence associated with the environment, the reward was secured during the training phase. Consequently, as illustrated in Equation (21), the implementation of Stochastic Gradient Ascent (SGA) within ADNet facilitated the necessary parameter updates for training the reinforcement learning model.


$$\begin{array}{c} {\Delta W}^{M_{RL}}\propto \sum_{L}^{\text{Env}}\frac{\partial \text{log}\left(p\left(M_{\text{RL}}\left(s_{L}\right)\right)\right)}{\partial W_{M_{\text{RL}}}}R_{L} \end{array}$$ (21)

## 4. Results

### 4.1 Evaluation details

(1) Evaluation metrics: The development of the RGBT tracking model, based on reinforcement learning and multimodal fusion techniques, aims to enhance robustness for UAVs in scenarios characterized by varied illumination and severe occlusions. This enhancement is intended to broaden the applicability of UAV target tracking tasks. The dataset included 60 experimental sequences, each assessed based on 12 attributes: NO, PO, HO, LI, LR, TC, DEF, FM, SV, MB, CM, and BC. PR and SR serve as the primary quantitative indicators to evaluate the effectiveness of the performance.

In target tracking tasks, PR quantifies the proportion of instances in which the computed position remains within a predefined proximity to the actual ground truth position. PR is determined by assessing the frequency at which the predicted location falls within an acceptable margin of the true value.


$$\text{PR}=\frac{\text{TP}}{\text{TP}+\text{FP}}$$ (22)

In the evaluation of tracking algorithms, SR is employed to measure the proportion of frames in which the overlap between the predicted and actual bounding boxes exceeds a specified threshold. SR is quantified by calculating the area under the curve, which aggregates these proportions across all frames.

(2) Experimental environment: In the established experimental setting, the system operated on Ubuntu 18.04. The hardware framework included an Intel i7-10700K processor, dual GeForce RTX 2080 Ti GPUs, and a memory capacity of 32GB. The development of all algorithms under consideration was conducted in Python, utilizing PyTorch as the deep learning platform.

### 4.2 Benchmark evaluation

The evaluation conducted on our self-constructed dataset illustrates the performance of our tracking algorithm across various attributes, with the leading performers for each attribute being highlighted as shown in Table 2. Our tracker achieves the highest overall performance with PR of 81.3% and SR of 58.2%. Unlike most trackers, which exhibit significant performance declines under conditions of occlusion, our method demonstrates robust resilience. Typically, trackers experience a pronounced decrease in performance during partial occlusion and severe occlusion conditions. However, our method, along with MANet++ and mfDiMP, maintains commendable PR results. In terms of both SR and PR, our tracker outperforms others, thereby maintaining high tracking effectiveness. This is attributed to our implementation of a tracking strategy based on dynamic template updates, which effectively mitigates tracking errors caused by changes in object shape and occlusion. Furthermore, under conditions of low illumination and low resolution, our tracker surpasses most existing tracking methods, including those like MANet++ and mfDiMP, which perform well in occlusion scenarios. This superior performance is primarily due to our technique’s capacity to fully harness and utilize the complementary data from RGB and thermal imaging, thereby underscoring the utility of multimodal information fusion. However, performance was suboptimal in thermal crossover scenarios, likely due to differences between synthetic and real RGB-T data. For attributes such as fast motion (FM) and motion blur (MB), the results are modest, impacted by limitations in offline training and local search capabilities. Nevertheless, our method excels in handling deformation (DEF) and scale variation (SV), achieving PRs of 81.9% and 82.7% and SRs of 61.3% and 60.6%, respectively. These results highlight its adaptability to changes in target appearance. Overall, the integration of multimodal fusion and dynamic template updates ensures consistent performance across diverse challenges, while maintaining a balance between accuracy and computational efficiency.

**Table 2:** Attribute-based precision and success rate (PR/SR) of different trackers on our self-constructed dataset. The top three trackers are highlighted in bold.

Method	SiamDW+	HDINet	DuSiamRT	SiamFT	SGT	MANet	MANet++	mfDiMP	Ours
NO	84.5/62.4	85.3/66.7	83.3/63.2	85.3/65.8	86.9/64.1	89.7/57.3	**90.7/66.9**	**90.5/66.8**	**90.6/68.2**
PO	74.7/55.8	75.3/56.2	78.3/57.9	82.4/**61.5**	76.7/53.4	78.9/54.3	**84.6**/58.6	**85.7/61.9**	**86.9/65.9**
HO	59.3/41.3	61.2/45.4	65.3/44.7	66.2/47.2	59.3/42.2	61.4/42.4	**71.8/48.9**	**71.6/48.5**	**71.9/49.5**
LI	61.0/43.4	62.3/44.5	65.2/47.3	66.3/47.5	70.2/47.4	73.4/48.5	**77.6/54.3**	**80.0/56.1**	**82.6/57.9**
LR	66.4/47.5	68.3/48.5	68.3/46.3	68.4/46.5	71.4/48.8	77.5/49.7	**78.7/54.5**	**77.9/52.3**	**78.3/54.8**
TC	73.2/52.1	73.6/55.2	**80.3/59.2**	**83.5/62.4**	72.7/52.7	**78.2**/49.4	77.3/**56.2**	77.5/54.7	72.4/49.8
DEF	70.7/53.2	71.7/55.3	72.3/54.1	75.5/56.3	71.5/55.9	**75.8/57.5**	74.0/54.8	**79.3/58.2**	**81.9/61.3**
FM	58.9/42.1	67.5/49.1	**72.5**/50.2	72.4/**50.5**	64.0/44.3	69.2/43.2	72.4/47.3	**75.8/53.2**	**73.4/50.4**
SV	74.3/55.9	74.9/57.5	80.6/59.4	**81.2/60.2**	74.3/52.9	71.5/46.3	79.3/56.2	**83.7/61.7**	**82.7/60.6**
MB	63.4/46.2	66.3/50.7	73.2/53.7	**74.4/55.3**	61.2/45.5	66.5/45.7	**74.5/53.8**	**76.4/54.3**	63.3/50.9
CM	66.2/49.2	68.3/51.9	68.3/50.4	72.4/54.3	65.4/48.7	68.2/47.2	**74.3/52.3**	**78.8/56.2**	**79.3/58.7**
BC	59.4/40.4	59.3/41.3	60.2/40.8	61.5/41.4	62.3/42.5	67.3/43.4	**74.9/51.6**	**73.3/49.5**	**75.3/53.5**
ALL	70.3/52.3	72.7/53.8	75.4/53.7	76.5/**56.2**	71.4/50.6	74.0/49.2	**79.3**/55.9	**80.6/56.3**	**81.3/58.2**

**Fig 8:**
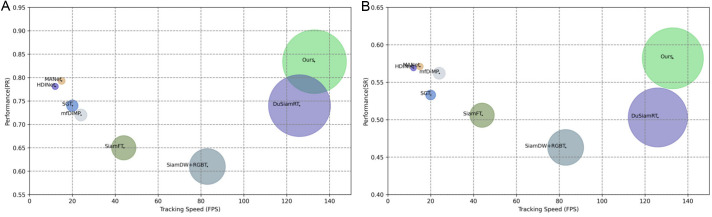
Speed comparison of various tracking methods. **(a)** PR and speed based on self-constructed dataset. **(b)** SR and speed based on self-constructed dataset.

In the subsequent analysis, we assessed the efficiency of SiamCMR in comparison to other fusion-based tracking methods, including SGT, mfDiMP [44], MANet [60], MANet++ [67], SiamDW [61] + RGBT, HDINet [62], DuSiamRT [63], and SiamFT [64], as depicted in Fig 8. SiamCMR achieved significantly higher speeds than most competing methods, reaching 135 FPS on our custom dataset. This model effectively balances robustness with processing speed through its utilization of dual-mode Siamese networks, which streamline the architecture. Additionally, methods like MWPM and CCFF were noted for their simplicity and user-friendliness compared to other comparative fusion techniques.

### 4.3 The ablation experiments

**Fig 9:**
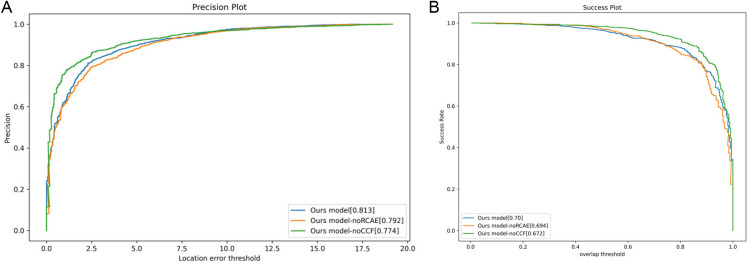
PR and SR comparison of the custom dataset. **(a**) PR comparison; **(b)** SR comparison.

Our initial ablation study was conducted using a custom dataset to evaluate the impact of critical components within SiamCMR. This analysis employed two altered versions of SiamCMR: the first, designated SiamCMR-noRCAE, omitted the residual channel attention enhancement module; the second, referred to as SiamCMR-noCCFF, excluded the CCFF module. The results were assessed based on the data presented in Fig 9.

SiamCMR demonstrated significantly superior PR/SR metrics compared to SiamCMR-noRCAE, indicating that the RCAE substantially bolstered the features of different modalities, thereby enhancing tracking performance. Furthermore, comparative assessments showed that SiamCMR consistently outperformed SiamCMR-noCCFF, confirming the CCFF’s ability to minimize inter-modal discrepancies through consistent feature extraction. This enhancement facilitated improved integration of visible and thermal infrared modalities. Moreover, experimental outcomes under challenging conditions, such as heavy occlusion, illumination variation, low illumination, and low temperatures, affirmed the effectiveness of the core components of SiamCMR.

### 4.4 Real-scene verification

**Fig 10:**
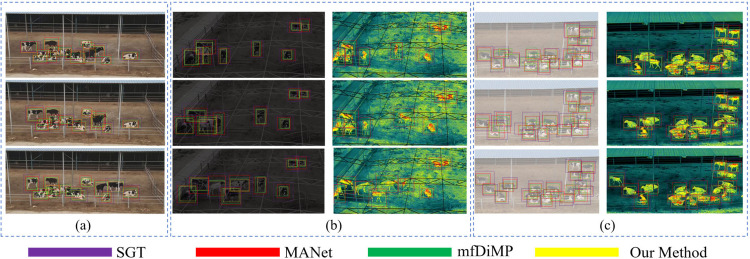
Visualization comparison between our method and other advanced trackers.

We executed real-scene tracking in environments characterized by severe occlusion (Fig 10a), low-light nighttime conditions (Fig 10b), and foggy circumstances with low resolution (Fig 10c). As depicted in Fig 10, the SGT tracker was unable to maintain tracking under severe occlusion, while the MANet tracker managed only partial tracking with low identification accuracy. In contrast, both our method and mfDiMP exhibited superior performance, particularly in managing severe occlusion cases. Our reinforcement learning-based approach facilitated the continuous retention of historical frame information, ensuring robust tracking even when the target was stationary due to occlusion or faced appearance alterations due to environmental or perspective changes. To further corroborate our method, we conducted tests under real-world conditions marked by insufficient illumination and occlusion (Fig 10b and 10c). These tests demonstrated that our tracker maintained stable performance, whereas SGT, MANet, and mfDiMP displayed lower precision and experienced more frequent tracking failures, particularly under occlusion. These findings demonstrating the effectiveness of multimodal information fusion.

## 5. Discussion

This study introduces SiamCMR, a sophisticated RGB-Thermal (RGBT) object tracking framework utilizing a dual-stream Siamese network architecture, specifically tailored for intelligent livestock monitoring in complex scenarios. The framework is particularly optimized for UAV applications, facilitating seamless deployment and operation on resource-limited edge computing platforms such as NVIDIA Orin NX. Through comprehensive ablation studies and field testing in open environments, the efficacy of two pivotal components has been substantiated: the CCFF module and MWPM. These components collaboratively enhance the integration of visible and thermal infrared modalities, thereby substantially augmenting tracking performance. Empirical evidence indicates that SiamCMR attains a PR of 81.3% at 135 FPS, marking a 4.8 percentage point increment relative to the baseline SiamFT method. Significantly, the incorporation of a reinforcement learning-based historical trajectory weighting mechanism elevates SR by at least 1.3% in scenarios characterized by PO and HO. This mechanism effectively mitigates prevalent challenges such as severe appearance changes, cluttered backgrounds, rapid target movements, and frequent occlusions, which have historically impeded monitoring accuracy in livestock management.

The present study has delineated two primary limitations concerning the application scope: (1) Scenario adaptability necessitates additional validation for environments with ultra-high density herds (exceeding 50 cattle per frame) and extreme motion blur conditions; (2) Target adaptability requires enhancement since the model’s specialized optimization for the distinctive black-and-white patterns of Holstein cows may hinder its generalization capabilities across monochromatic breeds. Building upon these insights, forthcoming research will concentrate on four strategic directions: the development of thermal image super-resolution networks [45], the coordination of UAV swarm tracking, the reduction of model size for enhanced efficiency, and the integration of multimodal biometric techniques. These initiatives aspire to actualize the concept of “unmanned grassland herd management” with cross-camera seamless tracking. The proposed innovations are poised not only to elevate the standards of livestock monitoring but also to serve as valuable references for other smart agriculture applications through the introduction of an innovative multimodal fusion framework.

## 6. Conclusion

This study addresses the challenges inherent in monitoring Holstein cows within intelligent livestock management systems by developing an RGBT tracking framework predicated on SiamCMR. The framework employs two distinct SiamCMR modules to independently extract features from RGB and thermal images. To enhance the efficacy of feature integration, a CCFF module, in conjunction with MWPM, is deployed to effectively fuse RGB and thermal data, thereby augmenting tracking reliability under adverse lighting conditions. Additionally, the incorporation of reinforcement learning techniques furthers the refinement of the system’s response to tracking perturbations caused by occlusions or drift. In environments marked by substantial occlusions, variable lighting, and suboptimal image quality, our approach attains state-of-the-art performance in terms of tracking accuracy, with success rates and precision reaching 81.3% and 58.2% respectively. Relative to the baseline tracker SiamFT, our methodology realizes significant enhancements, improving PR by 4.8 percentage points and SR by 2.0 percentage points. Moreover, it surpasses the current leading baseline, mfDiMP, with additional gains of 0.9 percentage points in PR and 1.9 percentage points in SR. Crucially, while maintaining superior accuracy, our approach also achieves a noteworthy 55% increase in processing speed compared to mfDiMP, thereby accomplishing a balanced optimization between tracking performance and real-time efficiency.

The implications of this study for the field of smart animal husbandry, particularly in terms of Holstein cow monitoring and management, are substantial. By developing an effective real-time cow-tracking method, this study elevates the cow-tracking system to a new level of operational excellence. This technological advancement significantly enhances management efficiency within dairy farming, empowering farmers and ranch managers to accurately monitor cow locations, behaviors, and health status. This, in turn, facilitates optimized feeding plans and improves production efficiency. Moreover, it provides robust support for ensuring the quality and safety of milk.
